# Rationale for vaccination with trivalent or quadrivalent live attenuated influenza vaccines: Protective vaccine efficacy in the ferret model

**DOI:** 10.1371/journal.pone.0208028

**Published:** 2018-12-03

**Authors:** Larisa Rudenko, Irina Kiseleva, Elena Krutikova, Ekaterina Stepanova, Andrey Rekstin, Svetlana Donina, Maria Pisareva, Elena Grigorieva, Kirill Kryshen, Arman Muzhikyan, Marina Makarova, Erin Grace Sparrow, Guido Torelli, Marie-Paule Kieny

**Affiliations:** 1 Department of Virology, Institute of Experimental Medicine, St Petersburg, Russia; 2 Department of Toxicology and Microbiology, Institute of Preclinical Research Ltd, St Petersburg, Russia; 3 Universal Health Coverage and Health Systems, World Health Organization, Geneva, Switzerland; 4 International Institutional Cooperation, Institut national de la santé et de la recherche médicale (INSERM), Paris, France; University of South Dakota, UNITED STATES

## Abstract

**Background and aim:**

The majority of seasonal influenza vaccines are trivalent, containing two A virus strains (H1N1 and H3N2) and one B virus strain. The co-circulation of two distinct lineages of B viruses can lead to mismatch between the influenza B virus strain recommended for the trivalent seasonal vaccine and the circulating B virus. This has led some manufacturers to produce quadrivalent influenza vaccines containing one strain from each B lineage in addition to H1N1 and H3N2 strains. However, it is also important to know whether vaccines containing a single influenza B strain can provide cross-protectivity against viruses of the antigenically distinct lineage. The aim of this study was to assess in naïve ferrets the potential cross-protective activity of trivalent live attenuated influenza vaccine (T-LAIV) against challenge with a heterologous wild-type influenza B virus belonging to the genetically different lineage and to compare this activity with effectiveness of quadrivalent LAIV (Q-LAIV) in the ferret model.

**Methods and results:**

Ferrets were vaccinated with either one dose of trivalent LAIV containing B/Victoria or B/Yamagata lineage virus, or quadrivalent LAIV (containing both B lineages), or placebo. They were then challenged with B/Victoria or B/Yamagata lineage wild-type virus 28 days after vaccination. The ferrets were monitored for clinical signs and morbidity. Nasal swabs and lung tissue samples were analyzed for the presence of challenge virus. Antibody response to vaccination was assessed by routine hemagglutination inhibition assay. All LAIVs tested were found to be safe and effective against wild-type influenza B viruses based on clinical signs, and virological and histological data. The absence of interference between vaccine strains in trivalent and quadrivalent vaccine formulations was confirmed. Trivalent LAIVs were shown to have the potential to be cross-protective against infection with genetically different influenza B/Victoria and B/Yamagata lineages.

**Conclusions:**

In this ferret model, quadrivalent vaccine provided higher protection to challenge against both B/Victoria and B/Yamagata lineage viruses. However, T-LAIV provided some cross-protection in the case of a mismatch between circulating and vaccine type B strains. Notably, B/Victoria-based T-LAIV was more protective compared to B/Yamagata-based T-LAIV.

## Introduction

Seasonal influenza A and B viruses circulate worldwide. However, circulation patterns and strain predominance vary widely from country to country and year to year. In the past decade, influenza H1N1 and H3N2 viruses have predominated, but influenza B viruses have recently become increasingly prominent [1, 2]. In the 1970s, influenza B viruses diverged into two major antigenically distinct lineages, B/Victoria and B/Yamagata [3, 4]. Since then, the two genetic lineages of influenza B virus—B/Victoria (B/Vic) and B/Yamagata (B/Yam)—are currently co-circulating among humans in various countries, one or the other lineage predominating in different areas [5–9].

According to recent estimates by the US Centers for Disease Control and Prevention (CDC) and the World Health Organization (WHO), up to 650 000 deaths a year are associated with seasonal influenza [10–12]. Annual vaccination is recommended to control influenza epidemics, and is especially important for those at high risk of severe disease and different vaccine types, injected and live, are currently available on the global market.

Live attenuated influenza vaccine (LAIV), produced by the Institute of Experimental Medicine (IEM), St Petersburg, Russia, has a long history of development and use. It has been used since 1987 for the prophylaxis of influenza in all age groups over three years old [13].

Since 1973, WHO has issued recommendations for the composition of seasonal influenza vaccines. Current vaccines are composed of one strain of H3N2, one strain of H1N1 and one B (trivalent influenza vaccine) or two B virus strains (quadrivalent influenza vaccine). If the influenza B virus strain recommended for the trivalent vaccine is not of the same antigenic lineage as the circulating virus, there may be increased influenza morbidity [14]. The lower effectiveness of such a mismatched vaccine could be avoided by including four virus strains in the vaccine—one from each B lineage in addition to H1N1 and H3N2. The use of quadrivalent influenza vaccine was suggested in the first decade of the 21^st^ century [15,16]. WHO first made recommendations for the composition of a quadrivalent influenza vaccine in September 2012 [17]. Quadrivalent LAIV (Q-LAIV) is licensed in US (since 2012), Canada (since 2013) and European Union (since 2015) and in a limited number of other countries. As of today, only one Q-LAIV is licensed: FluMist (MedImmune, AstraZeneca). However, in a number of countries including Russia only trivalent LAIV (T-LAIV) is used.

The wide cross-protectivity of live influenza vaccine virus has been previously described [18] and recent studies in ferrets have suggested that natural infection with one influenza B virus lineage may be provide protection against subsequent infection with either influenza B virus lineage [19]. It would therefore be useful to have more evidence on the cross-protectivity of Russian T-LAIV containing one lineage of influenza B virus to the other B lineage virus.

This study first assessed the potential cross-protective activity of Russian trivalent LAIV (T-LAIV) in naïve ferrets against challenge with heterologous wild-type (WT) influenza B virus belonging to a genetically different lineage. The secondary objective was to compare this activity with the protection provided by quadrivalent LAIV (Q-LAIV).

## Materials and methods

### LAIVs tested

Two trivalent and one quadrivalent LAIVs were tested. All three LAIVs contained vaccine candidates against A/New York/61/2015 (H1N1)pdm09 strain which is A/Michigan/45/2015 (H1N1)pdm09-like virus, and A/Hong Kong/4801/2014 (H3N2) strain. One T-LAIV (B/Vic) also contained B/Texas/02/2013 strain, which is B/Brisbane/60/2008-like virus (Victoria lineage), while the second T-LAIV (B/Yam) contained B/Phuket/3073/2013 strain (Yamagata lineage). The Q-LAIV (B/Vic, B/Yam) contained both B/Phuket/3073/2013 strain (Yamagata lineage) and B/Texas/02/2013 strain (Victoria lineage). Titers of the individual components in the trivalent and quadrivalent preparation ranged from 7.1 to 7.8 lg EID_50_ per dose. The T-LAIV and Q-LAIV were prepared and supplied by the Serum Institute of India (Pune, India). All LAIV reassortant strains included in the vaccine preparations were developed at IEM on the base of Russian master donor viruses, A/Leningrad/134/17/57 (H2N2) and B/USSR/60/69, except B/Texas/02/2013 strain (Victoria lineage), which was generated by the back-up laboratory facility at CDC on the base of Russian master donor virus B/USSR/60/69.

### Animals and husbandry

Female ferrets, 5–6 months of age and weighing 0.7–1.1 kg at the beginning of the experiment, were supplied by Scientific Production Organization “House of Pharmacy JSC” (St Petersburg, Russia). They were prescreened with a routine hemagglutination inhibition (HAI) test to ensure that they were negative to circulating human influenza viruses, and to the viruses being tested. The following viruses were used as antigens for prescreening HAI: (i) A/New York/61/2015 (H1N1)pdm09 strain; (ii) A/Hong Kong/4801/2014 (H3N2) strain; (iii) B/Phuket/3073/2013 strain (Yamagata); and (iv) B/Texas/02/2013 strain (Victoria). The animals were housed and handled in accordance with European Union legislation [20]. At the end of the study, the animals were euthanized with a combination of Zoletil and Xylazine. All experimental procedures were taken in accordance with the international guidelines on the ethical treatment of laboratory animals [20]. The animal use protocol #0037–17 was approved on April 18, 2017, by the Local Bioethical Committee of the Institute of Preclinical Research Ltd (St Petersburg, Russia).

On day 0, groups of three ferrets each were immunized intranasally with 1.0 ml of T-LAIV (B/Vic) (groups 1 and 2), T-LAIV (B/Yam) (groups 3 and 4) or Q-LAIV (group 5 and 6). Three additional groups of three ferrets each (groups 7, 8 and 9) were given 1.0 ml of phosphate-buffered saline (PBS). Preparations were divided over the two nostrils and given under short-term anesthesia induced by intramuscular injection of Zoletil 100 at a dose of 12.5 mg/kg of body weight.

**Fig 1:**
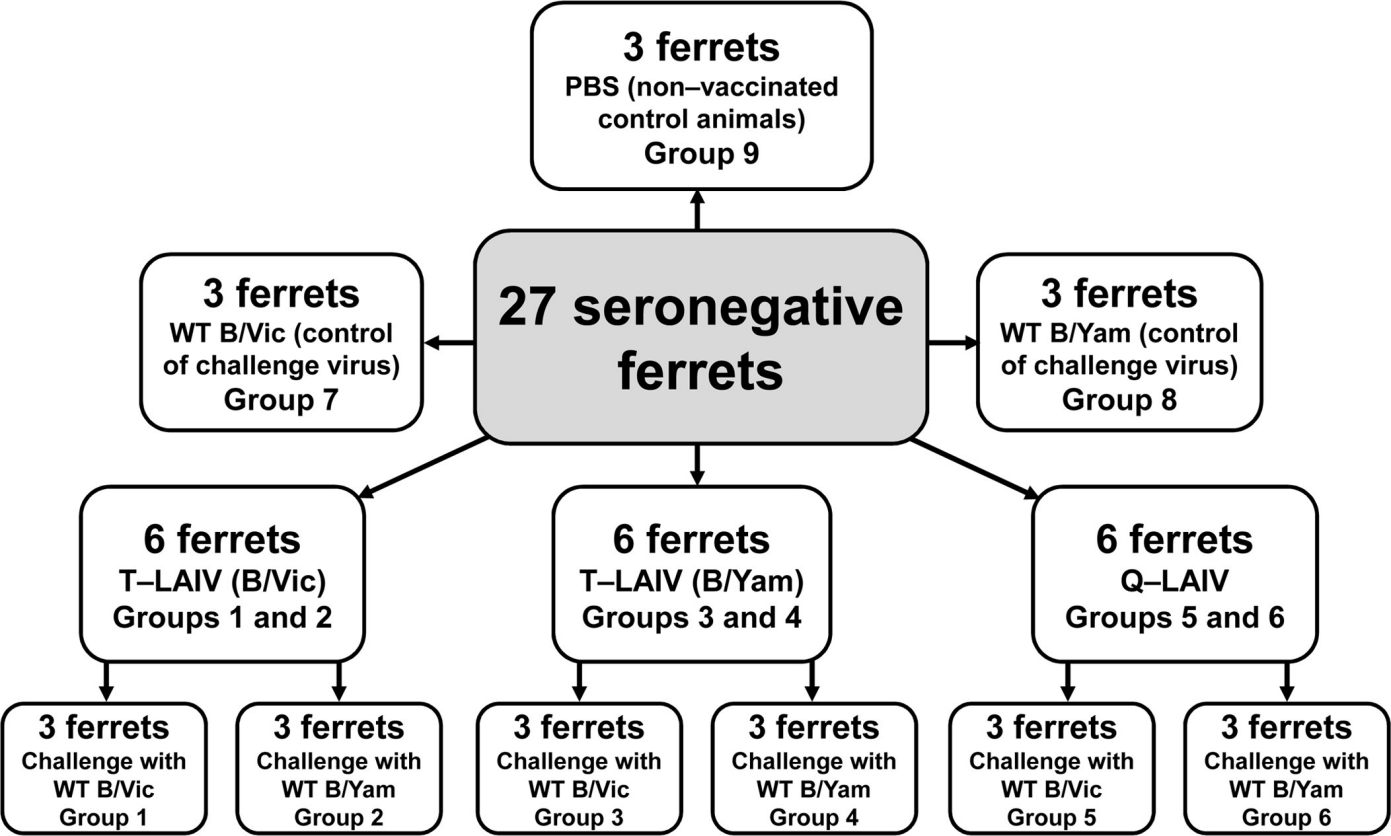
Test groups of ferrets.

Four weeks after immunization (day 28), ferrets in groups 1, 3, 5 and 7 were challenged with 1.0 ml of 6.0 lg EID_50_/ml B/Brisbane/60/2008 (Victoria lineage) WT virus. Those in groups 2, 4, 6 and 8 were challenged with 1.0 ml of 6.0 lg EID_50_/ml B/Phuket/3073/2013 (Yamagata lineage) WT virus (Fig 1). Blood samples for serum preparation were collected 2 days prior to vaccination. Post vaccination blood samples were collected 28 days later.

### Monitoring for clinical signs and morbidity

Ferrets were monitored closely for three days following vaccination and for three days following challenge. Clinical signs were assessed; ferrets were evaluated for nasal symptoms and level of activity. Nasal symptoms were scored as follows: 1—nasal rattling could be heard or the ferret sneezed during transport from its cage to the evaluation area; 2—there was evidence of nasal discharge on the external nares; 3—animals exhibited mouth breathing; 0—the animal exhibited none of these symptoms. Ferret activity was scored over a range from 0 to 3 depending on the extent to which the animal could be induced to play: 0—the animal was fully playful; 1- the animal responded to play overtures but did not initiate any play activity; 2—the animal was alert but not at all playful; 3—the animal was neither playful nor alert. Results are presented as a sum of scores per group.

The body temperature of the animals was recorded by temperature loggers (DST micro-T ultra-small temperature logger; Star-Oddi, Reykjavik, Iceland), which were placed in the peritoneal cavity.

### Virus isolation in embryonated chicken eggs

Nasal washes were collected for detection of viral shedding three days after vaccination and challenge. Samples of lung tissue were taken three days after challenge (day 31) and analyzed for the presence of challenge virus by titration in embryonated chicken eggs. Nasal washes were tested by inoculation of 10-fold dilutions in 10-11-day-old embryonated chicken eggs (Nazia poultry plant, St Petersburg, Russia) and incubation at 32°C for 72 hours. The presence of influenza virus was detected by standard hemagglutination (HA) test with 1% chicken red blood cells (RBCs) [21].

### Virus detection by polymerase chain reaction

Nasal secretions collected three days after vaccination and challenge were tested by real-time polymerase chain reaction (RT-PCR) for detection of influenza B virus RNA and determination of virus subtype. RNA extraction from the nasal washes was performed using “RIBO-sorb” reagent kit for RNA/DNA isolation from human specimens (InterLabService, Central Research Institute of Epidemiology under Rospotrebnadzor, Moscow, Russia). RT-PCR testing was performed using SuperScript III Platinum One-step RT-qPCR System (Invitrogen). Primers and Taqman probes for influenza B viruses (B/Yam and B/Vic) RNA amplification test were provided by CDC.

The RT-qPCR titer was calculated as described in [22] using Rotor-Gene 1.8.17.5 Software on the basis of the set values of EID_50_ titers of individual B components of T-LAIVs and Q-LAIV used in the experiment. The set values indicated for each vaccine component served as calibrators. The calibration line was automatically constructed (FAM channel) for: 100 dilutions of the vaccine preparations in 3 replicates. The calculation was performed for each vaccine component separately (R^2^≥0.98). The RT-qPCR titer in 1.0 ml of nasal washes or lung suspension calculated for each HA gene target (B/Yam and B/Vic) corresponds the vaccine component EID_50_ virus titer.

### Hemagglutination inhibition

HAI assays were performed before the study and after vaccination. The serum antibody response to vaccination was measured by standard HAI test [21] with 1% chicken RBCs using 4 hemagglutinin units (HAU) of antigen. Serum samples were pretreated with receptor destroying enzyme (RDE, Denka Seiken, Tokyo, Japan). The final dilution factor of the sera after RDE treatment was 1:10. Titers < 1:10 in the first sera taken before vaccination were set to “5” for analysis. A fourfold or more rise in antibody titer after vaccination (titers ≥ 1:20) was considered a reliable indicator of seroconversion. The following antigens were used for HAI assay: (i) A/New York/61/2015 (H1N1)pdm09 strain; (ii) A/Hong Kong/4801/2014 (H3N2) strain; (iii) B/Phuket/3073/2013 strain (Yamagata); and (iv) B/Texas/02/2013 strain (Victoria).

### Gross pathology

A complete macroscopic post-mortem (gross pathology) examination was performed on all animals. The trachea and lungs were studied in detail, and the abdominal and pelvic cavities were examined. All lung lobes were inspected. Macroscopic changes in the lungs were assessed in points in accordance with the color, the number of foci and the severity of the lesion. After necropsy, lungs were collected and weighed.

### Histopathology

Tissue sections of trachea and lungs were taken. One lobe of the lungs from each sacrificed animal was analyzed histologically, while the other lobe was used to study viral reproduction. Material for the histological study was fixed in 10% neutral formaldehyde solution for 24 hours, then according to the standard technique it was embedded in paraffin, and the histological sections 3–5 μm thick were prepared. Tissue sections were stained with alcian blue at pH 2.5 with finish staining with hematoxylin and eosin. Lung tissue damage analysis was performed for following three main categories and hislological parameters—epithelial damage (exudate lumen bronchi, hypertrophy bronchi epithelium, hyperplasia bronchi epithelium, necrosis bronchi epithelium, exudate lumen bronchioli, hypertrophy bronchiolar epithelium, hyperplasia bronchiolar epithelium, necrosis bronchiolar epithelium), inflammation (bronchitis, peribronchitis, bronchiolitis, peribronchiolitis, perivasculitis, vasculitis, interstitial infiltrate, alveolitits) and alveolar damage (hyperemia septa, alveolar emphysema, alveolar hemorrages).

Histological parameters were semi-quantitatively scored in accordance with the system of Widjojoatmodjo [23]: 0 = absent; 1 = minimal; 2 = slight; 3 = moderate; 4 = strong; and 5 = severe. Morphological study was made blinded for 3 sections from each lung using the light optic microscope Axio Scope A1 (Carl Zeiss, Germany) with integral digital camera AxioCam ICc 1 (Carl Zeiss, Germany) and AxioVision Rel. 4.8 program (Carl Zeiss, Germany).

### Statistics

The Shapiro-Wilk test was used to assess distribution parameters (normality test). Differences between groups were analyzed statistically using one-way analysis of variance (ANOVA), post-hoc Tukey test, Kruskal-Wallis ANOVA by ranks, median test, Student's t-test or Mann-Whitney U test by Statistica 10.0 (StatSoft, USA). Differences were considered significant at *P* ≤ 0.05.

## Results

### Vaccine safety

#### Body weight

Table 1 shows the body weight change of ferrets from day 0 to day 3 post vaccination. The same increase in body weight was seen in the groups that received vaccine as in the control groups (groups 7, 8 and 9). Thus, immunization with T- or Q-LAIV did not affect the body weight.

**Table 1:** Clinical observations in ferrets vaccinated with trivalent or quadrivalent LAIV and challenged with B/Brisbane/60/2008 (B/Vic) or B/Phuket/3073/2013 (B/Yam) wild-type influenza viruses.

Vaccine (group)	Сlinical signs^1^				Body weight relative to day 0 post				Lung examination^3^
					vaccination/day 28 post challenge (%)^2^				
**After vaccination**	Day 0	Day 1	Day 2	Day 3	Day 0	Day 1	Day 2	Day 3	Day 3
Non-vaccinated ferrets (groups 7, 8 and 9)	0(0;0)	0(0;0)	0(0;0)	0(0;1)	100	100.8 ± 0.38	99.9 ± 1.03	99.3 ± 1.16	n.d.^4^
T-LAIV (B/Vic) (groups 1 and 2)	0(0;0)	0(0;1)	0(0;1)	0(0;1)	100	98.0 ± 0.64	96.5 ± 0.84	95.7 ± 1.23	n.d.
T-LAIV (B/Yam) (groups 3 and 4)	0(0;1)	0(0;0)	0(0;1)	1(0;2)	100	100.8 ± 0.63	98.8 ± 0.81	97.9 ± 1.20	n.d.
Q-LAIV (groups 5 and 6)	0(0;1)	0(0;0)	1(0;1)	0.5(0;1)	100	99.1 ± 0.61	97.9 ± 0.97	96.8 ± 1.36	n.d.
**After challenge with B/Vic**	Day 28	Day 29	Day 30	Day 31	Day 28	Day 29	Day 30	Day 31	Day 31
Non-vaccinated ferrets (group 9)	0(0;0)	0(0;0)	0(0;0)	0(0;0)	100	98.0 ± 0.67	95.7 ± 0.34	96.0 ± 0.69	12.7 ± 1.76
T-LAIV (B/Vic) (group 1)	0(0;0)	0(0;0)	0(0;0)	0(0;0)*	100	97.3 ± 1.87***	96.4 ± 1.40***	96.7 ± 0.68***	16.3 ± 2.73
T-LAIV (B/Yam) (group 3)	0(0;0)	0(0;0)	0(0;1)	0(0;1)*	100	94.4 ± 1.06	93.6 ± 1.27	93.3 ± 1.15	22.0 ± 2.08
Q-LAIV (group 5)	0(0;0)	0(0;0)	0(0;0)	0(0;0)*	100	94.1 ± 0.80	94.1 ± 0.56***	94.9 ± 0.51	19.3 ± 2.73
Control (group 7)	0(0;0)	0(0;1)	1(0;3)	4(2;4)	100	94.2 ± 0.19	90.2 ± 1.36	90.1 ± 2.48	35. 7 ± 1.76****
**After challenge with B/Yam**	Day 28	Day 29	Day 30	Day 31	Day 28	Day 29	Day 30	Day 31	Day 31
Non-vaccinated ferrets (group 9)	0(0;0)	0(0;0)	0(0;0)	0(0;0)	100	98.0 ± 0.67	95.7 ± 0.34	96.0 ± 0.69	12.7 ± 1.76
T-LAIV (B/Vic) (group 2)	0(0;0)	0(0;0)	0(0;0)	0(0;0)**	100	94.9 ± 1.45	94.8 ± 1.41	94.0 ± 1.71	18.7 ± 2.91
T-LAIV (B/Yam) (group 4)	0(0;0)	0(0;3)	0(0;0)	0(0;0)**	100	96.0 ± 0.71	94.7 ± 0.10	94.7 ± 1.28	17.3 ± 2.03
Q-LAIV (group 6)	0(0;1)	0(0;1)	0(0;0)	0(0;0)**	100	93.6 ± 3.18	93.4 ± 2.76	93.3 ± 1.69	19.0 ± 3.21
Control (group 8)	0(0;0)	0(0;1)	1(0;1)	2(1;3)	100	94.5 ± 1.10	93.7 ± 1.13	94.7 ± 1.26	32.7 ± 2.73****

^1^Sum of scores, median (first quartile; third quartile).

^2^Semi-quantitative analysis of body weight, average weight per ferret ± standard error of mean (M ± SEM)

^3^Semi-quantitative analysis of lung tissue, average sum of scores per ferret ± SEM.

^4^not detected.

*Significantly different from control group 7 by Kruskal-Wallis ANOVA by ranks (*P* = 0.025), median test (*P* = 0.029), Mann-Whitney U test *P* values: 0.034 (group 1 vs. 7); 0.043 (group 3 vs. 7); 0.034 (group 5 vs. 7).

**Significantly different from control group 8 by Kruskal-Wallis ANOVA by ranks (*P* = 0.013), median test (*P* = 0.007), Mann-Whitney U test *P* values: 0.037 (group 2 vs. 8); 0.037 (group 4 vs. 8); 0.037 (group 6 vs. 8)

***Significantly different from control group 7 by Mann-Whitney U test (*P* = 0.050).

****Significantly different from non-vaccinated ferrets of group 9 by one-way ANOVA, post-hoc Tukey test, group 7 *P* = 0.004, group 8 *P* = 0.010.

#### Clinical signs

Table 1 shows the scores for clinical signs. On days 1–3 after immunization, a reduction in overall activity of up to 1 point was noted. Only one ferret immunized with T-LAIV (B/Yam) showed decreased activity with a score of 2 points on day 3. The behavior of the vaccinated animals (groups 1–6) did not differ from that in the control groups (7, 8 and 9). No clinical signs of infection were detected in ferrets vaccinated with T- or Q-LAIV.

#### Body temperature

**Fig 2:**
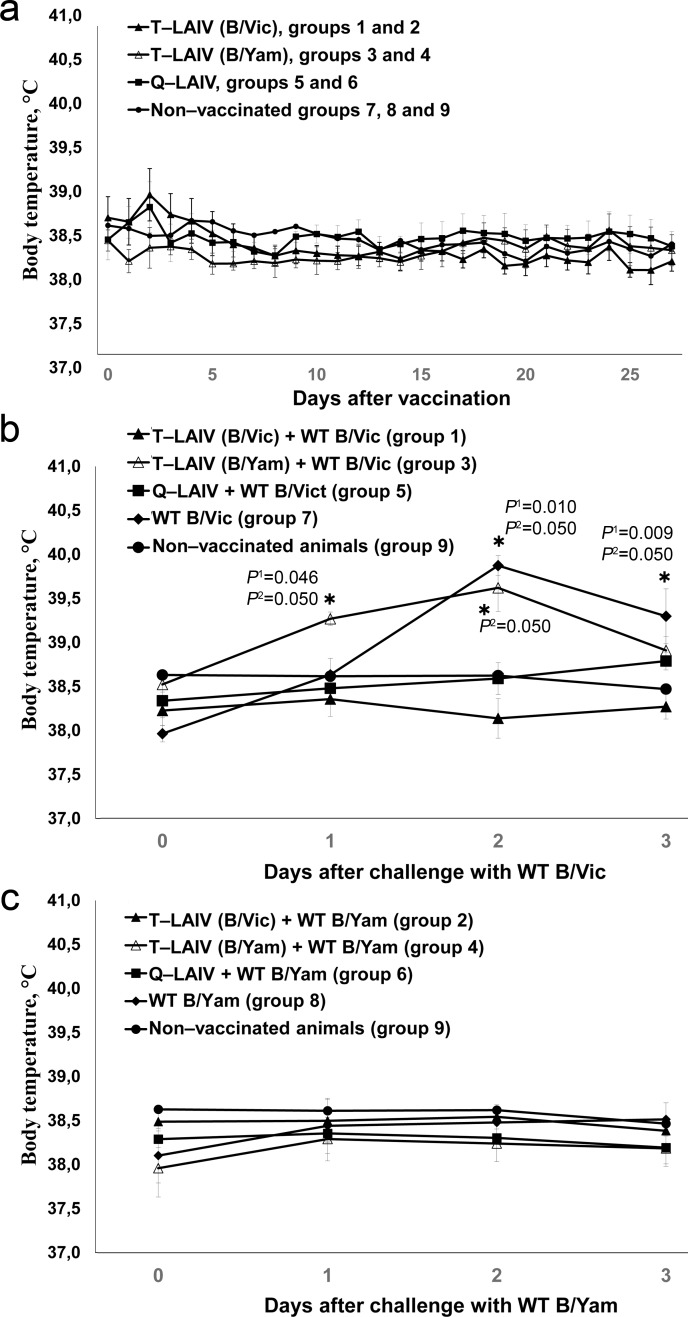
Body temperature of ferrets. (a) after vaccination with T-LAIVs, Q-LAIV or PBS; (b) after challenge with WT B/Vic following by vaccination with T-LAIVs, Q-LAIV or PBS; (c) after challenge with WT B/Yam following by vaccination with T-LAIVs, Q-LAIV or PBS. *significantly different from non-vaccinated, non-challenged group (*P*^1^: Kruskal-Wallis ANOVA (all groups); *P*^2^: Mann-Whitney U test (vs. group 9)).

The body temperature of the ferrets on days 0–27 is shown in Fig 2A. One-way ANOVA showed no statistically significant effects of vaccine administration on body temperature in any day.

#### Vaccine viral replication

The presence of vaccine virus in the upper respiratory tract of the ferrets was confirmed by titration of nasal washes. The total virus titer on day 3 after vaccination was sufficient and varied from 4.20 to 4.53 lg EID_50_/ml (Table 2).

**Table 2:** Isolation of vaccine viruses from nasal washes of ferrets in embryonated chicken eggs on day 3 post vaccination.

Vaccine	Group	Vaccine virus titer
		(lg EID_50_/ml, Mean ± SEM)
		(No. isolated/total)
T-LAIV (B/Vic)	1 and 2	4.53 ± 0.33 (6/6)
T-LAIV (B/Yam)	3 and 4	4.38 ± 0.12 (6/6)
Q-LAIV	5 and 6	4.20 ± 0.12 (6/6)
Non-vaccinated ferrets	7, 8 and 9	< 1.5 (0/9)

Pronounced replication of each vaccine virus in the upper respiratory tract was confirmed by real-time PCR. The titer of different components of vaccine virus in nasal washes on day 3 post-vaccination was close to 4 RT-qPCR/ml (Table 3).

**Table 3:** Vaccine virus shedding on day 3 and humoral immune response to components of LAIV on day 28 post vaccination, respectively.

Vaccine	Day 3 post vaccination				HAI GMT (day 0/day 28)			
	Vaccine virus titer in nasal washes, RT-qPCR/ml, Mean ± SEM				(twofold dilutions)			
	H1N1	H3N2	B/Vic	B/Yam	H1N1	H3N2	B/Vic	B/Yam
T-LAIV (B/Vic)	3.350 ± 0.865	3.376 ± 0.968	4.409 ± 0.899	< 1.5	5.0 / 452.5	5.0 / 201.6	5.0 / 35.6	5.0 / 5.0
T-LAIV (B/Yam)	4.832 ± 0.493	4.379 ± 0.274	< 1.5	4.912 ± 0.568	5.0 / 806.3	5.0 / 127.0	5.0 / 5.0	5.0 / 31.7
Q-LAIV	4.012 ± 0.308	3.727 ± 0.279	4.633 ± 0.312	4.892 ± 0.314	5.0 / 570.2	5.0 / 71.3	5.0 / 17.8	5.0 / 22.4
Non-vaccinated ferrets	< 1.5	< 1.5	< 1.5	< 1.5	5.0 / 5.0	5.0 / 5.0	5.0 / 5.0	5.0 / 5.0

### Vaccine protection

#### Body weight

Table 1 shows the body weight change of the ferrets from day 0(28) to day 3(31) after challenge. The data follow a normal distribution. The body weight of the vaccinated ferrets after challenge with wild-type B influenza viruses (groups 1–6) did not differ significantly from that of the non-vaccinated ferrets (group 9). A minimum weight loss was seen in the group 1 immunized with the T-LAIV containing B/Victoria vaccine strain after challenge with homologous WT B/Vic virus (about 3%). There were no significant intergroup differences. The greatest reduction in body weight, of more than 6%, was recorded in the control animals inoculated with WT challenge viruses, B/Vic and B/Yam (groups 7 and 8, respectively).

#### Clinical signs

The scores for clinical signs after challenge with WT virus are presented in Table 1. On day 31, unvaccinated ferrets (groups 7 and 8) had lower overall activity and showed symptoms of the respiratory system in the form of coughing. The activity of individual animals was evaluated as sluggish. The general condition of animals in groups 1–6 after challenge was satisfactory.

#### Body temperature

The body temperature of the ferrets was recorded on days 28–31 (Fig 2B). A significant rise in body temperature (1.5–2°C, average 40°C) was seen in the unvaccinated animals after challenge with wild-type influenza virus B/Victoria (group 7) compared with the control group 9 (Fig 2B). This indicates the development of disease in these animals.

The ferrets immunized with T-LAIV (B/Vic) or Q-LAIV showed no significant increase in body temperature after challenge with B/Victoria, compared with control group 9. In addition, statistically significant differences in body temperature were seen in the groups of ferrets immunized with T-LAIV containing B/Victoria and the control animals (group 7) on day 3(31) after challenge with B/Victoria virus (Mann-Whitney U test, P = 0.050). These results suggest that a single immunization with T-LAIV (B/Vic) or Q-LAIV is effective against B/Victoria challenge.

In contrast, based on body temperature measuring ferrets of group 3 vaccinated with T-LAIV (B/Yam) did not display statistically significant difference compared with control group 7 (WT B/Vic) on day 2(30) (*P =* 0.658, group 3 vs. group 7, Mann-Whitney U test).

Challenge of ferrets with WT B/Yam had no effect on body temperature on days 29–31. However, ferrets of control group 8 also did not displayed increase in body temperature on days 1–3 post infection.

#### Assessment of viral replication by end-point titration in eggs

**Fig 3:**
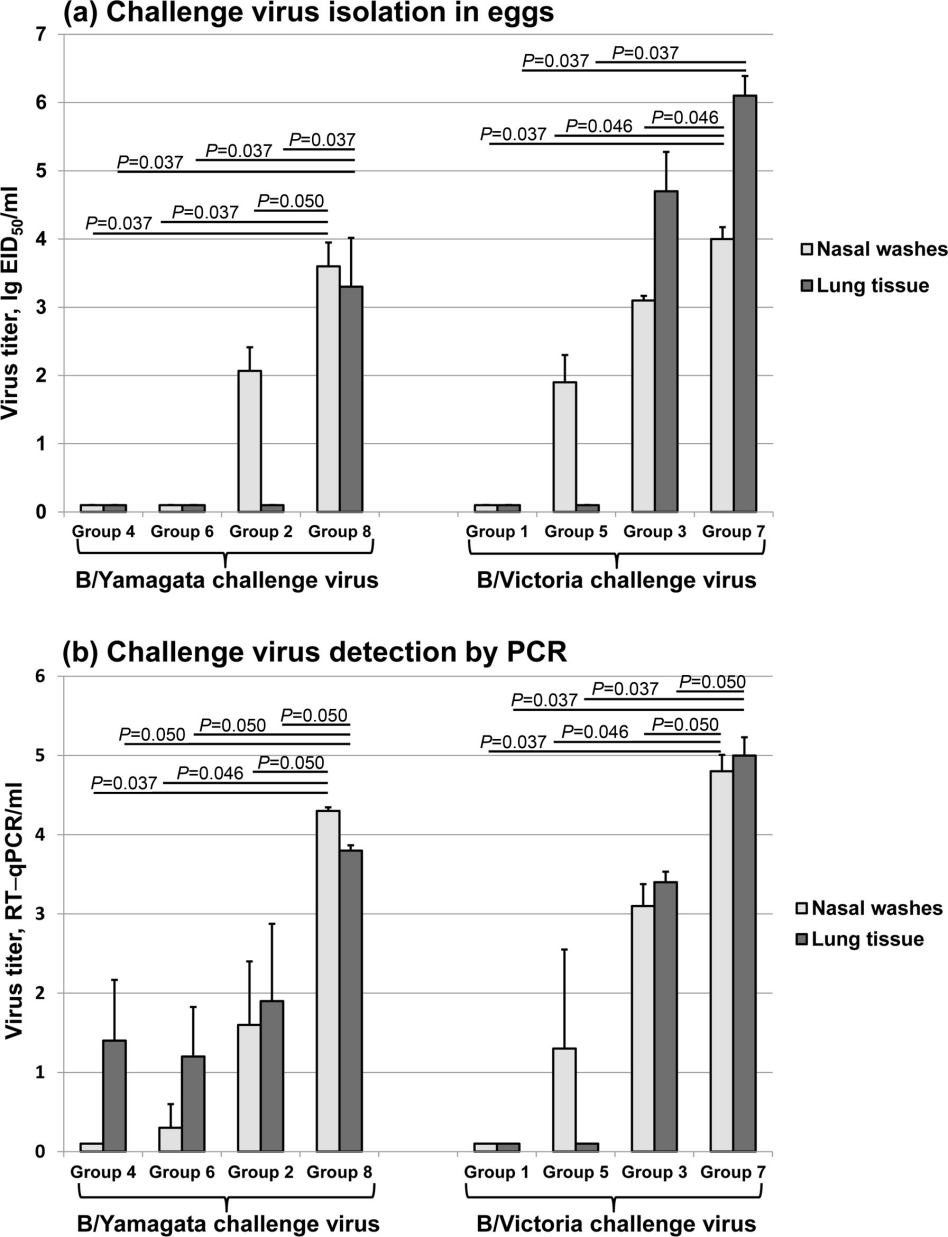
Detection of challenge virus in ferrets. (a) detection of challenge virus by culturing test samples in embryonated eggs; (b) detection of challenge virus in test samples by quantative RT-PCR. *P* values (Mann-Whitney U test) are stated. T-LAIV (B/Vic) + WT B/Vic (group 1); T-LAIV (B/Vic) + WT B/Yam (group 2); T-LAIV (B/Yam) + WT B/Vic (group 3); T-LAIV (B/Yam) + WT B/Yam (group 4); Q-LAIV + WT B/Vic (group 5); Q-LAIV + WT B/Yam (group 6); WT B/Vic (control of challenge virus, group 7); WT B/Yam (control of challenge virus, group 8).

B/Yamagata challenge virus was not detected in the nasal washes or lungs of any animal that received T-LAIV containing B/Yamagata or Q-LAIV. Surprisingly, a significantly lower replication of B/Yamagata in nasal washes was found in ferrets vaccinated with T-LAIV containing B/Victoria compared with the control group 8 (2.1 lg EID_50_/ml vs. 3.6 lg EID_50_/ml, Mann-Whitney U test, *P* = 0.0495) (Fig 3A). There was also no replication of virus in the lungs of ferrets vaccinated with T-LAIV containing B/Victoria.

B/Victoria challenge virus was not detected in the lungs of any animal that received T-LAIV containing B/Victoria or Q-LAIV. A significantly lower replication of B/Victoria was found in nasal washes of ferrets vaccinated with Q-LAIV (1.9 lg EID_50_/ml) than in the control group 7 (4.0 lg EID_50_/ml) (Mann-Whitney U test, *P* = 0.046) (Fig 3A). In ferrets that received T-LAIV containing B/Yam, B/Victoria challenge virus replication in the upper respiratory tract was significantly lower than in the control group but not so pronounced as it was seen in ferrets that received T-LAIV containing B/Vic after challenge with WT B/Yamagata virus (3.1 lg EID_50_/ml vs. 4.0 lg EID_50_/ml, Mann-Whitney U test, *P* = 0.046). Replication of B/Victoria challenge virus in the lungs in this group was not significantly different than in the control group (4.7 lg EID_50_/ml vs. 6.1 lg EID_50_/ml, Mann-Whitney U test, *P* = 0.127) (Fig 3A).

These data demonstrate that vaccination with T-LAIV (B/Vic) or T-LAIV (B/Yam) may protect ferrets against homologous and heterologous challenge with type B virus of different genetic lineages. In particular, vaccination inhibits replication of both homologous and heterologous challenge virus in upper respiratory tract. T-LAIV containing B/Victoria had a more pronounced effect on the replication of the heterologous challenge virus in low respiratory tract compared with T-LAIV (B/Yam). Lung replication of WT B/Vic in group 3 given T-LAIV (B/Yam) was not statistically significant decreased (*P* = 0.127, Mann-Whitney U test). However, the tendency to reduce the B/Vic challenge virus titer can be seen on Fig 3A.

#### Assessment of viral replication by real-time PCR

Significantly lower replication of both homologous and heterologous challenge virus in the upper respiratory tract and lungs of ferrets vaccinated with T- or Q-LAIV than in the control group was confirmed by real-time PCR (Fig 3B). B/Victoria challenge virus was not detected in the lungs of any animal that received T-LAIV containing B/Victoria or Q-LAIV.

Replication of B/Yamagata challenge virus in the lungs was significantly lower for all vaccinated ferrets than for the control group (Mann-Whitney U test, *P* = 0.0495) (Fig 3B).

The results obtained were found to be close to those obtained by virus isolation in eggs.

#### Assessment of serum antibody response by HAI assay

In the prescreening, all ferrets were seronegative to the viruses to be tested. Specifically, all the ferrets had an HAI antibody titer < 1:10 to B/Brisbane/60/2008 (Victoria lineage) or B/Phuket/3073/2013 (Yamagata lineage). For calculation of the geometric mean titer (GMT), the < 1:10 value was replaced with the absolute value 5. On day 28, the majority of vaccinated ferrets showed a 4-fold or greater increase in HAI antibody titer to the homologous influenza virus (Table 3). The highest increases in GMT were observed for the H1N1pdm09 component of the LAIVs (90.5–161.3-fold). Smaller changes were noted for the H3N2 component (14.3–40.3-fold). The lowest antibody titer increases were recorded for the B components (3.6–7.1-fold).

In contrast, antibody titers to the heterologous B virus were not detected. None of the ferrets vaccinated with B/Victoria had HAI antibody titers to WT B/Yamagata challenge virus (GMT = 5). Equally, none of those vaccinated with B/Yamagata virus had HAI antibody titers to WT B/Victoria challenge virus (Table 3).

#### Interference between vaccine components

There was pronounced replication of all A and B components of T- and Q-LAIV in the upper respiratory tract of each vaccinated ferret. On day 3 (the peak of replication), the vaccine virus titers as measured by real time PCR in all vaccinated groups 1–6 were close to 4 RT-qPCR/ml (Table 3).

The B/Victoria component of T-LAIV induced an antibody response close to that induced by the B/Yamagata component of T-LAIV (GMT = 35.6 for B/Victoria and 31.7 for B/Yamagata). Antibody titers in ferrets given Q-LAIV were slightly lower (GMT = 17.8 for B/Victoria and 22.4 for B/Yamagata). There was no statistically significant difference in the immune responses to the B-components of T- and Q-LAIV (Student t-test, for B/Victoria t = 1,18, *P* = 0,06; for B/Yamagata t = 0,96, *P* = 0,50).

#### Gross pathology

The histopathology results from samples of organs taken from the vaccinated groups were similar to those obtained from the control group 9. Administration of the LAIV vaccines did not cause significant gross morphological changes (S1 Fig).

Challenge of LAIV-vaccinated animals with wild type virus did not result in serious abnormal changes in lung tissue. Thus, LAIVs provided protection from lung infection with wild type influenza virus in ferrets.

#### Histopathology

**Fig 4:**
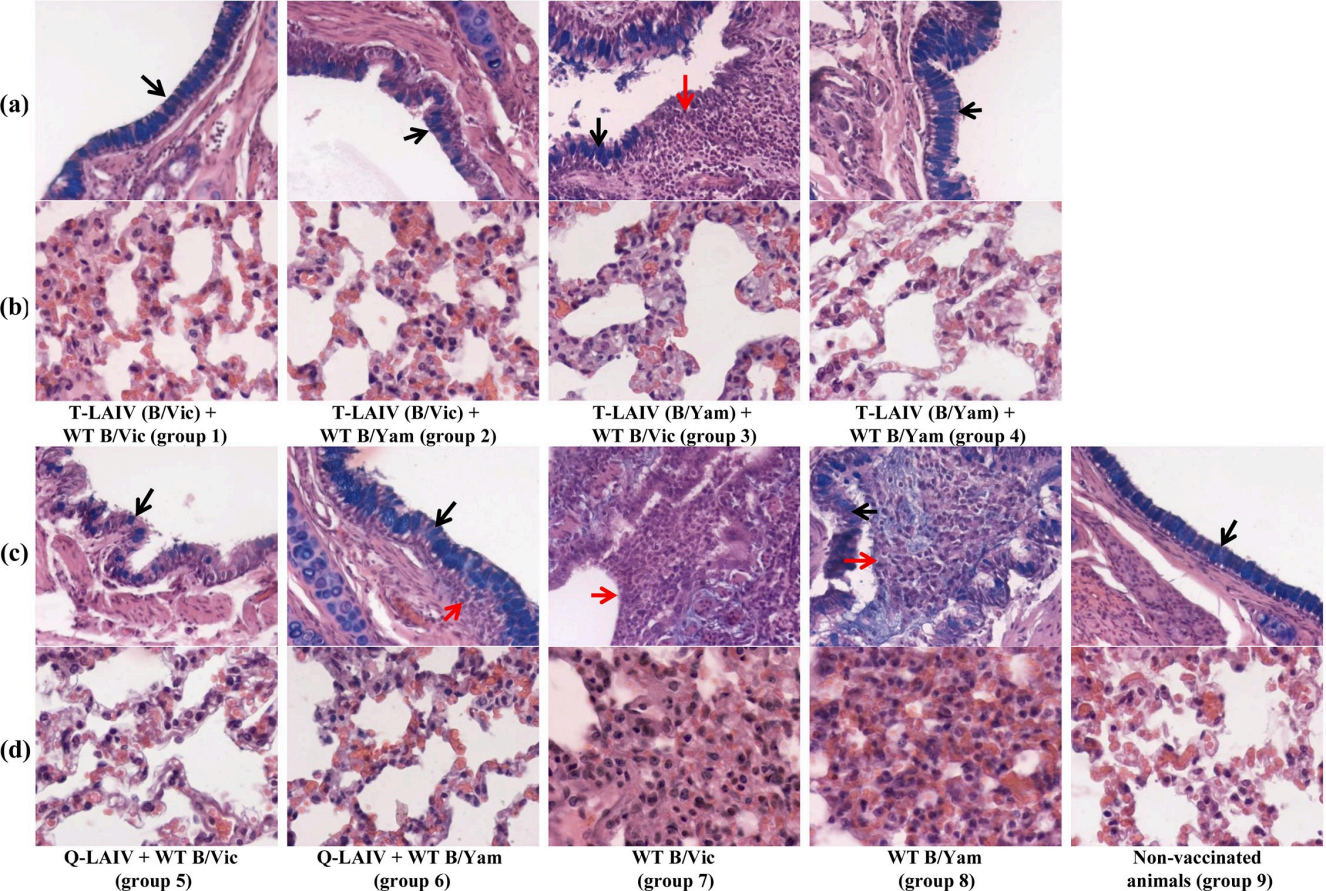
Histological view of the lungs. Red arrows indicate inflammation; black arrows indicate presence of goblet cells. (a) Bronchus. Alcian blue, H&E x 200; (b). Alveolar tissue. Alcian blue, H&E x 400; (c) Bronchus. Alcian blue, H&E x 200; (d). Alveolar tissue. Alcian blue, H&E x 400.

Microscopic examination of the tracheas did not reveal any pathological changes. The histological structure of the trachea of all examined animals was normal. Macroscopic and histological examination of the lung tissue of control ferrets of group 9 showed very little or no pathological changes (S1 Fig and Fig 4). The average scores per group (± standard error of mean) are presented in Table 1.

In the unvaccinated ferrets challenged with wild-type virus (groups 7 and 8), the mean scores for histopathological changes in the lungs were statistically significantly higher than in the unchallenged group. The lungs of ferrets infected with wild-type B/Victoria strain had macroscopic firm consolidated areas and emphysema foci of varying severity. Exudate was observed in the lumen of the bronchi and bronchioles, in which lymphocytes and mononuclear cells predominated. Mild to moderate signs of catarrhal bronchitis, peribronchitis, bronchiolitis, perivasculitis and vasculitis were seen. Damage to pulmonary tissue was observed in the form of expressed lymphocytic and mononuclear infiltration in the interstitial tissue of the respiratory department, hyperemia of alveolar septa and alveolitis, manifested as thickening of alveolar septa, diffuse inflammatory infiltration and hyperplasia of type 2 pneumocytes. Focal hemorrhages and alveolar emphysema were also seen. A characteristic feature was a markedly lower number of goblet cells in the bronchial epithelium in animals of this group compared with other groups. The mean score for lung lesions was 35.7 compared with 12.7 in the control group 9 (Tukey test, *P* = 0.004).

Ferrets challenged with B/Yamagata showed morphological changes characterized by injuries such as thickening and hyperplasia of the epithelium of the large and small bronchi, often with a microscopic picture of catarrhal bronchitis and bronchiolitis. Exudate was detected in the lumen of the medium-caliber bronchi and bronchioles, with a predominance of lymphocytes and mononuclear cells. Occasionally, there was necrotic damage to the bronchial epithelium, accompanied by moderate to severe lymphocytic infiltration. The mean score was 32.7 compared with 12.7 in the control group 9 (Tukey test, *P* = 0.010).

All groups of LAIV-vaccinated animals showed fewer pathological changes after challenge with WT virus than the unvaccinated groups, with very little or no inflammation. The epithelium of the large bronchi was only slightly changed, except in group 3. The alveoli showed no pathological changes, and the alveolar epithelium had oxyphilic cytoplasm and clear nuclei. The epithelium of the bronchi was unchanged. There were no acute inflammatory changes in the lung tissue. The mean scores were between 16.3 and 22.0, compared with 12.7 in the control group 9 (Table 1). Ferrets in group 3 (B/Yamagata vaccine and B/Victoria challenge) had fewer goblet cells of bronchial epithelium compared with the control group 9. In two animals, slight damage to the bronchial epithelium was found, comprising a low degree of lymphocytic and mononuclear infiltration in the interstitial tissue of the respiratory system. The mean score for this group was 22.0.

## Discussion

Trivalent seasonal influenza vaccine contains one strain of influenza B; however, it is not always possible to predict which influenza B lineage will predominate during the next influenza season [15, 16, 24–26]. There has often been a mismatch between vaccine components and the most prevalent epidemic influenza viruses, which may affect vaccine efficacy [8, 25, 27, 28].

To try to circumvent this problem, several manufacturers have developed quadrivalent inactivated influenza vaccines, including Sanofi Pasteur, Green Cross, GlaxoSmithKline, SK Chemicals and Seqirus [29]. A quadrivalent LAIV was licensed in the USA in 2012 by MedImmune and is now in use in the USA, Canada and some European countries [30]. The main argument in favor of use of quadrivalent vaccines is the above mentioned potential mismatch between the influenza vaccine B component and the major epidemic B virus strain. However, development and use of Q-LAIV in different countries is impacted by a number of factors, such as increased production costs and time, vaccine price and cost-effectiveness, regulatory authorities’ requirements, vaccine supply policies etc. In this regard the use of Q-LAIV remains controversial, and a number of pro and contra opinions and questions regarding use of Q-LAIV have been raised. Many papers have discussed the impact of moving from trivalent to quadrivalent vaccines and discussed issues of cross-protection, cost-effectiveness, increased reactogenicity and interference between components [15, 16, 19, 26, 27, 30–44].

Q-LAIV has been less studied than quadrivalent inactivated vaccine. In Russia, Q-LAIV has yet to be investigated. The safety of the MedImmune Q-LAIV among children, adolescents, and adults has been demonstrated [26, 30, 36]. No clinically meaningful adverse events were associated with the administration of Q-LAIV in people aged between 2 and 49 years. Thus, including an additional vaccine component does not increase the reactogenicity of LAIV.

Clearly, matched strains provide the best protection. However, little is known about the protective efficacy of unmatched vaccine strains, especially for LAIV. Evidence of cross-protection of unmatched LAIVs is still contradictory and sparse.

Children are the most sensitive age group to influenza B viruses. However, in immunologically naïve children, vaccination with subunit monovalent B/Yamagata vaccine strain did not induce detectable hemagglutination-inhibiting or neutralizing antibody to B/Victoria-like viruses [45]. The authors concluded that the best option was to include strains of the two B lineages in influenza vaccines. Conversely, vaccination with subunit trivalent vaccine containing B/Victoria strain induced a significant rise in HAI antibody titer against B/Yamagata in middle-aged and elderly volunteers [46]. These data indicate a potential for cross-protection.

Ferrets have been successfully used to model human influenza virus infection since 1930s when the virus was first isolated. This model is thought to most accurately represent human influenza disease [47–50]. One of the main clinical manifestations of influenza infection in ferrets is an increase in body temperature. The normal body temperature of ferrets ranges from 37.8°C to 40°C (average 38.8°C). According to the literature, infection with influenza virus leads to a significant rise in body temperature in ferrets of 1–1.5°C, but patterns may differ greatly in individual animals and depending on the type of virus. Another important clinical signs of disease in ferrets following infection with influenza virus are development of respiratory symptoms and decline in body weight [47, 50–52].

In this study, a single dose of T-LAIV or Q-LAIV had no adverse effect on the body weight, body temperature or clinical condition of ferrets. The general condition of the vaccinated animals was close to normal. As assessed by body temperature, infection of non-vaccinated ferrets with wild-type influenza B/Victoria led to the development of disease. Immunization of animals with T-LAIV (B/Vict), or Q-LAIV proved to be effective against B/Victoria challenge. In contrast, the wild-type B/Yamagata strain itself was less reactogenic in ferrets than the B/Victoria WT strain. A significant rise in body temperature was not seen in the animals of non-vaccinated group 8 after inoculation with wild-type influenza virus B/Yamagata compared with the control group 9. Hence, challenge of ferrets with WT B/Yam had no effect on body temperature on days 29–31.

Unvaccinated ferrets inoculated with WT viruses showed symptoms of respiratory disease and reduced overall activity. In contrast, when vaccinated animals were challenged, their general condition, body temperature, and post mortem examination were close to normal. A single immunization of ferrets with T- or Q-LAIV followed by infection of these animals with either homologous or heterologous wild-type challenge virus has led to a significant reduction of clinical manifestation of viral infection. The overall efficiency of protection was good.

Ferrets given PBS (non-vaccinated control animals of group 9) showed an insignificant decline in body weight of about 2%, which was possibly due to the general anesthesia.

Clinical indicators of protection in vaccinated animals were more pronounced in groups vaccinated with T-LAIV containing B/Victoria; however in animals vaccinated with T-LAIV containing B/Yamagata lineage virus and challenged with heterologous B/Victoria virus their body weight and temperature were not significantly different from those of unvaccinated control animals.

One dose of T- or Q-LAIV led to production of specific HAI antibodies to the vaccine virus (GMT changes varied from 4-7-fold for B/Victoria to 161-fold for H1N1pdm09); despite antibody response to influenza B virus was not very high ferrets were protected from homologous challenge infection as shown by clinical signs and replication of challenge virus. The absence of correlations between LAIV-mediated protection and low antibody titer was confirmed both experimentally and in clinical trials. Early publications on the correlates of immune protection against influenza justified a lower antibody post-vaccination protective titers (1:10 and 1:20) being associated with protection of adults against influenza B infection [53–55]. The protection conferred by the LAIV could be attributed not only to the antibody response but also to the cell-mediated and/or local mucosal immune responses, particularly in naive ferrets [56]. Early studies served as a basis for WHO to formulate the idea that HAI assay is not appropriate for measuring immune responses to LAIV and LAIV immunity is the result of a combination of different types of immune responses [57–58].

In this study a cross-lineage antibody response to hemagglutinin was not detected. This finding corresponds with that of Belshe et al. [31], who demonstrated an absence of cross-reacting serum antibodies against the mismatched lineage of influenza B in ferrets. Nevertheless, our results showed that T-LAIV has the potential to be cross-protective against infection with genetically different influenza B lineages. Previous experiments in mice have demonstrated that IgA in nasal secretions may provide cross-protection against challenge infection with heterologous influenza B lineage virus [59]. An *in vitro* study confirmed that virus-specific polyclonal CD8+ cytotoxic T-lymphocyte populations obtained from human leukocyte antigen (HLA)-typed healthy study subjects cross-react with heterologous influenza B lineage virus [60]. These data indicate the potential for cross-protection against antigenically distinct lineages of influenza B viruses through local and cellular immune response.

In unvaccinated ferrets inoculated with WT viruses, pronounced virus replication was found in the upper and lower respiratory tract. In contrast, vaccinated animals challenged with the homologous WT virus had no virus replication. Interestingly, there was some cross-protection; ferrets vaccinated with T-LAIV containing B/Victoria had a lower level of B/Yamagata challenge virus in the upper respiratory tract than unvaccinated animals, and no challenge virus was detected in the lungs.

Ferrets vaccinated with T-LAIV containing B/Yamagata vaccine virus also had a lower level of B/Victoria challenge virus in the upper and lower respiratory tracts than unvaccinated animals. However, in this case, cross-protection was less pronounced. This finding is in line with the clinical data of other authors. Skowronski et al. [35] reported that annual vaccination of children with trivalent inactivated influenza vaccine containing B/Victoria strongly recalled serum antibodies to B/Yamagata. On the other hand, immunologically naïve children were not protected against infection with a B/Victoria strain after vaccination with B/Yamagata [45].

Interference between strains is one of major problems of virus multicomponent vaccines. However, in this study T- and Q-LAIV vaccine preparations seemed to be well balanced in respect of a possible interference between the three or four different vaccine virus strains, respectively. Despite relatively low antibody titers to B Yamagata- and Victoria-lineage components of T- and Q-LAIV, a good correlation was found with corresponding titers after vaccination of ferrets with monovalent LAIV. Antibody response to monovalent LAIV was similar (B/Vic) or slightly higher (B/Yam) when compared with T- and Q-LAIV, correspondingly. In particular, T-LAIV containing B/Victoria induced an antibody response (GMT = 35.6) close to that induced by the Q-LAIV (GMT = 17.8) and mono LAIV (B/Vic) (GMT = 35.6). B/Yamagata T-LAIV induced a response (GMT = 31.7) close to that induced by Q-LAIV (GMT = 22.4). Monovalent LAIV (B/Yam) provided a slightly higher response (GMT = 89.8) [61].

Our results confirmed the absence of interference between vaccine strains in T- and Q-LAIV based on Russian master donor viruses. B/Victoria and B/Yamagata strains were equally robust and did not suppress the immune response to the other B component. This finding corresponds to those of other authors [19, 32, 37].

## Conclusions

Both T- and Q-LAIV were found to be safe and effective against wild-type influenza B viruses, as indicated by clinical signs and virological and histological data. There was no interference between vaccine strains in trivalent and quadrivalent vaccine formulations.

T-LAIVs have the potential to be cross-protective against the genetically different influenza B lineages, possibly via immune mechanisms other than humoral immune response, for instance, cross-reactive secretory IgA antibodies or cross-reactive T-cell-mediated immunity may be responsible for cross-protection.

Vaccination with T-LAIV containing B/Victoria had a more pronounced effect on replication of B/Yamagata challenge virus than vice versa.

In this ferret model, quadrivalent vaccine provided higher protection to challenge against both B/Victoria and B/Yamagata lineage viruses. However, T-LAIV provided some cross-protection in the case of a mismatch between circulating and vaccine type B strains. Notably, B/Victoria-based T-LAIV was more protective compared to B/Yamagata-based T-LAIV.

## Supporting information

S1 Fig Macroscopic view of the lungs of ferrets. White arrows indicate hemorrhage; asterisks indicate focal emphysema.
(TIF)
