# Supplementary figures and images for: Rationale for vaccination with trivalent or quadrivalent live attenuated influenza vaccines: Protective vaccine efficacy in the ferret model
Source: PLoS One. 2018 Dec 3;13(12):e0208028. doi: 10.1371/journal.pone.0208028 (PMC6277076; doi:10.1371/journal.pone.0208028)

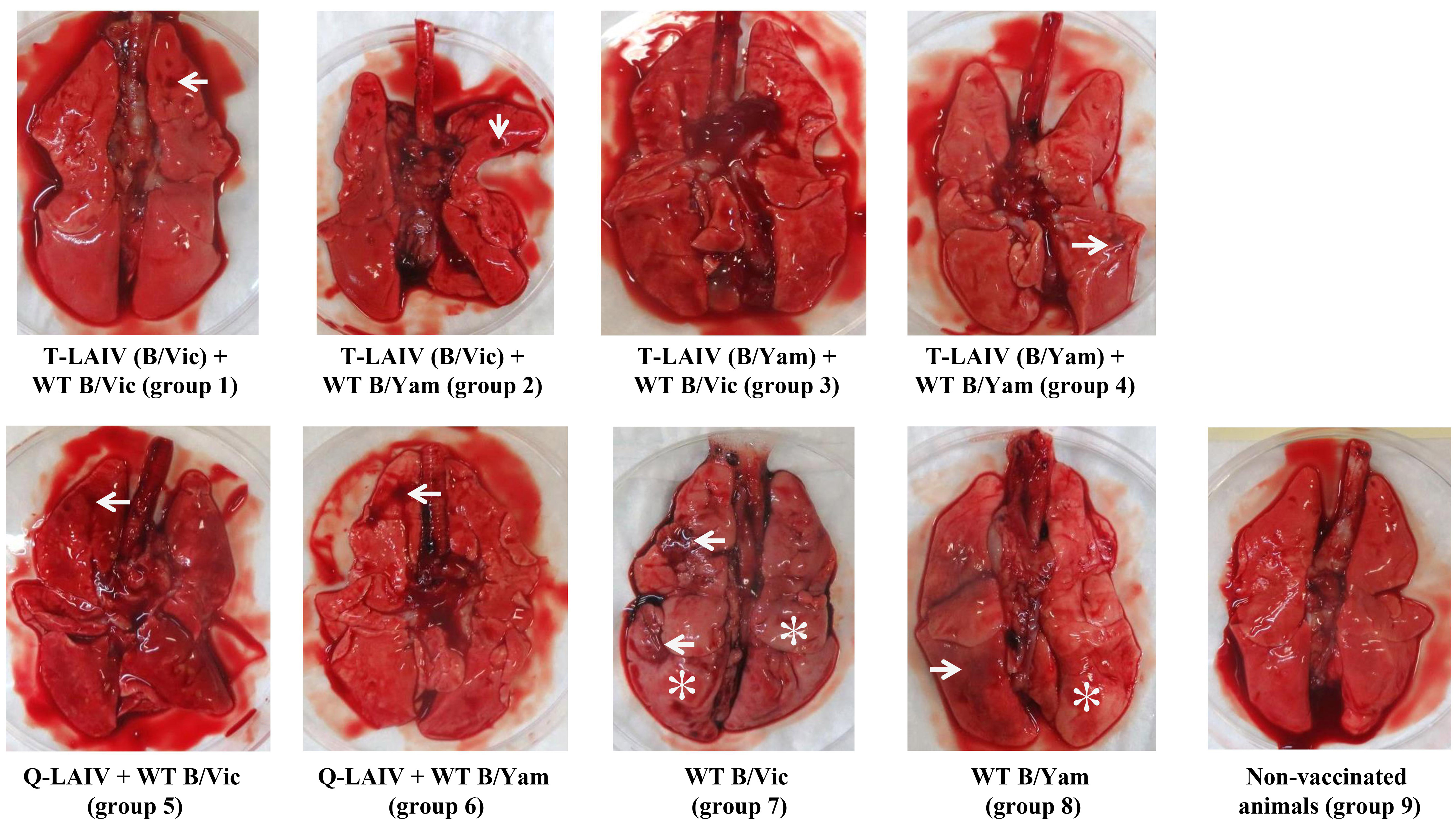

Supplement: S1 Fig — White arrows indicate hemorrhage; asterisks indicate focal emphysema. (TIF) [file pone.0208028.s001.tif]
